# 

**Published:** 1896-02

**Authors:** 


### INDEX TO VOLUME XII.

ORIGINAL COMMUNICATIONS—

Atlanta Medical College Clinics (Bell) ........ 726
Acute Croupous Pneumonia, Treatment of (Cain) ........ 217
Anesthetics, The Administration of (Morgan) ........ 85
Address at Southern Medical College (Harris) ........ 513
"Cancer" of the Skin, The Practical Treatment of (Hutchins) ........ 193
Contusions and Sprains of the Back, with Special Reference to the Early Treatment of these Injuries (Wharton) ........ 79
Coal-Tar Derivatives (Duncan) ........ 17
Case of Tetanus Affecting the Muscles of Jaws only (Mitra) ........ 400
Curious Cases of Atropia Poisoning (Mitra) ........ 660
Diphtheria, The Antitoxin Treatment of (Barker) ........ 13
Diagnosis and Medical Treatment of Intestinal Obstruction (Williams) ... 654
Eclampsia after Labor (York) ........ 481
Gunshot Wounds, Some Remarks on (McHatton) ........ 89
Hemorrhage after Tonsil Operations, the Management of (Calhoun) ........ 340
History of Medicine and Surgery in Georgia, (Grandy) ........ 65
Intracapsular Fractures of the Neck of the Femur (Baxter) ........ 583
Laparotomy During Pregnancy (Hardon) ........ 257
Lesions Associated with Dysmenorrhea (Gardner) ........ 599
Local Application of Cold in Acute Pneumonia (Mays) ........ 647
Laryngeal Stenosis (Bell)) ........ 724
Morphinomania, Mental Suggestions as an aid in the Treatment of (Green) ........ 140
Medical Examinations for Life Insurance Companies (West) ........ 7
Medical Aspect of Trephining in Epilepsy (Foster) ........ 449
Management of Labor in Cases of Minor Pelvic Deformity (Allen) ........ 385
Nasal Obstructions and Catarrh (Tinsley) ........ 10
Opium Curse, and a Prevention (Happel) ........ 270
Obstetrical Practice, Modern (Hurt) ........ 144
Onychogryphosis, with Cornu Cutaneum, A Case of (Inghram) ........ 75
Placenta Previa (Guntermann) ........ 20
Pneumothorax (Allen) ........ 392
Physiology of the Bile and Gall-Bladder, and Etiology of Cholelithiasis (Levy) ........ 402
Pneumonia (Hurt) ........ 641
Penetrating Wounds of Abdomen (Winslow) ........ 718
Report of Part of my Surgical Work for the Six Months ending April 13th, With Remarks (Holmes) ........ 129 223
Report of Two Cases from Notes Found in my Record Book, with Remarks (Mitchell) ........ 149

Remarks on Goitre, with Report of a Case (White).... 593
Rickets (Shaw)... 466
School Children's Eyes, The Necessity for Proper Care of (Roy)... 207
Spastic Paralysis: Talipes Equino-Varus (Owen)... 1
Specialties in Medicine and Surgery, Rudimentary Preparation Compared with Higher Education for (Gaston)... 200 278
Surgical Cases, Report of (Armstrong)... 321
Surgical Treatment of Cholelithiasis (Taylor)... 528
Surgical interference in Rectal Disorders (Gaston)... 577
Symmetrical Gangrene, Raynaud's Disease (Hutchins)... 722
Therapeutics of Heart Disease (Wood)... 472
Treatment of Cutaneous Cancers (Robinson)... 713
Uric Acid as a Factor of Disease (Van Goidtsnoven)... 535
Visual Fields and Fundous Changes in Retinitis Pigmentosa (Stirling)... 328
Vital Statistics, etc,, and the Hygiene of the Convicts, Hospital and Prison Camps of the Penitentiary of Georgia, from October 1, 1890, to October 1, 1894 (Daniel)... 336
What Shall be Done with the Homicidal Crank (Diller)... 405
Zoster (Gottheil)... 264

SOCIETY REPORTS—

Allegheny County Medical Society... 411
Atlanta Society of Medicine... 24 287 541 729 662
Cincinnati Obstetrical Society... 343
Medical Association of Georgia... 153
Richmond Academy of Medicine and Surgery... 27 93 238
Tri-State Medical Society... 548 602

CORRESPONDENCE—

Chemical Action of Quinine, Strychnine and Atropine... 419
Department of Public Health... 611
Marion County (Fla.) Medical Society... 676
Malarial Hematuria... 608
New York Letters... 241 291 350 671 482
Pudendal Hematocele... 293
Radical Treatment of Diseases of Nose and Throat... 487
Removal of Mesoblastic Tumor of Neck... 738
Sage Tea for Hiccough... 100
Treatment of Hemorrhoids by Iodoform in Ether... 30
Ten Rules Governing Administration of Ether... 610

EDITORIALS—

A National Board of Health... 32
Are We Drifting in Our Ethics?... 356
Amik *vs.* Reeves... 357
A "Qualifyde" Practitioner on Boards, Colleges, etc... 740
Bicycling for Women... 421
Bogus Medical Journalism... 742

EDITORIALS—CONTINUED.

Commencements.......... 105
Cotton States and International Exposition.......... 423
Dr. Robert Battey.......... 489 613
Dr. Thomas S. Powell.......... 682
Dr. Jas. E. Reeves.......... 741
Examinations by the Regular Board of Medical Examiners, April 4, 1895 170
Execution by Electricity.......... 557
Fakir Journalism.......... 490
Hypnotism and Crime.......... 169
Insanity, Medical Expert Testimony and the Courts.......... 425
Louis Pasteur.......... 556
Ministerial Indorsements of Humbugs.......... 34
Medicine as She is Learned.......... 491
The Medical Association.......... 102
Orificial Surgery.......... 298
Southern Surgical and Gynecological Association.......... 615
Status of Antitoxin.......... 677
The Meeting in Savannah.......... 167
The Late Meetings in Baltimore.......... 241
The Christian Advocate and the Keely Cure.......... 250
The Passing of the Antitoxin.......... 295
The late Professor Huxley.......... 358
The Journal's Premiums.......... 683
Volume Twelve.......... 32

SELECTIONS AND ABSTRACTS—

Eye, Ear, Nose, and Throat.......... 35 108 182 299 427 559 688 744
General Medicine and Therapeutics.......... 39 175 304 363 501 618 685 749
Miscellaneous.......... 691 767
Obstetrics and Gynecology.......... 50 117 436 763
Practical Chemistry and Pharmacy.......... 379 432
Skin Diseases and Syphilis.......... 44 106 360 497 625
Surgery.......... 56 113 438 493 565 627 747

BOOK REVIEWS..........59 122 187 252 313 382 445 509 570 638 708 775

MEDICAL ITEMS ..........63 127 190 255 318 381 443 507 570 635 704 769

### CONTRIBUTORS TO VOLUME XII.

---

| | |
|---|---|
| Armstrong, W. S., M.D | Atlanta. |
| Allen, Jos. Eve, M.D | Augusta. |
| Allen, Chas. L., M. D | New York. |
| Barker, L. F., M.D | Baltimore. |
| Bell, W. J. M.D | Atlanta. |
| Bell, R. W | Atlanta. |
| Baxter, G. A., M.D | Chattanooga. |
| Cain, J. S., M.D | Nashville. |
| Calhoun, A. W., M.D | Atlanta. |
| Diller, Theodore, M.D | Pittsburg. |
| Duncan, J. W., M.D | Atlanta. |
| Foster, Eugene, M.D | Augusta. |
| Gunterman, P., M.D | Louisville. |
| Grandy, Luther B., M.D | Atlanta. |
| Green, Saml. H., M.D | Oakdale. |
| Gaston, J. McFadden, M.D | Atlanta. |
| Gottheil, Wm. S., M D | New York. |
| Gardner, Wm. S., M.D | Baltimore. |
| Holmes, J. B. S., M.D | Atlanta. |
| Hurt, C. D., M.D | Atlanta. |
| Hutchins, M. B., M.D | Atlanta. |
| Hardon, Virgil O., M.D | Atlanta. |
| Happel, T. J., A.M., M.D | Trenton, Tenn. |
| Hutchins, M. B.. M.D | Atlanta. |
| Hurt, C. D., M.D | Atlanta. |
| Harris, Henry F., M.D | Atlanta. |
| Inghram, W. H., M.D | Atlanta. |
| Levy, H. H., M.D | Richmond. |
| Morgan, Jas. B., M D | Augusta. |
| McHatton, H., M.D | Macon. |
| Mitchell, T. Emerson, M.D | Columbus. |
| Mays, Thos. J., M.D | Philadelphia. |
| Mitra, N. C., M.A., M.B | Bengal, India. |
| Owen, Mr. Edmon | London. |
| O'Daniel, W., M.D | Bullard. |
| Robinson, A. R., M.D | New York. |
| Roy. Dunbar, A.B., M.D | Atlanta. |
| Stirling, Alex. W., M.D | Atlanta. |
| Shaw, Chas. S., M.D | Pittsburg. |
| Tinsley, Austin S., M.D | Augusta. |
| Taylor, Hugh M., M.D | Richmond. |
| Van Goidtsnoven, E., M.D | Atlanta. |
| West, Jas. N., M.D | Toccoa. |
| Wharton, Henry R., M.D | Philadelphia. |
| Winslow, Randolph, M.D | Baltimore. |
| Williams, R. F., M.D | Richmond. |
| White, Jos. A., M.D | Richmond. |
| Wood, H. C., M.D | Philadelphia |
| York, D. A., M.D | Cornelia. |

NOTICE.—Our offer of a thermometer to *all* cash-in-advance subscribers is withdrawn. *New* subscribers, paying full year in advance, may still get the thermometer. Book offer still open to all who pay a year in advance. "Antisepsis and Antiseptics," "Stories of a Country Doctor."

